# Profiles of Cognitive Appraisal of Non-Suicidal Self-Injury Scars and Their Clinical Correlates in Adults with a History of Self-Injury: A Cross-Sectional Study

**DOI:** 10.3390/ejihpe16100161

**Published:** 2026-10-10

**Authors:** Alexandrina A. Grigoreva, Ilya A. Fedotov, Maksim P. Marachev, Anastasia K. Kondratovich

**Affiliations:** 1 Department of Pedagogy and Medical Psychology, I.M. Sechenov First Moscow State Medical University ( Sechenov University), 8-2 Trubetskaya Street, 119991 Moscow, Russia; marachev_m_p@staff.sechenov.ru; 2 Department of Clinical Psychology, Ryazan State Medical University Named After Academician I.P. Pavlov, 9 Vysokovoltnaya Street, 390026 Ryazan, Russia; ilyafdtv@yandex.ru; 3 Neurocentre of Medical-Psychological Correction and Rehabilitation, 3-1 Severny Boulevard, 127566 Moscow, Russia; 4 Department of Psychoprophylaxis, V. Serbsky National Medical Research Centre for Psychiatry and Narcology, 23 Kropotkinskiy Pereulok, 119034 Moscow, Russia; kondratovich.a@serbsky.ru

**Keywords:** non-suicidal self-injury, scars, cognitive appraisal, shame, suicidal ideation, psychotherapy, cluster analysis

## Abstract

Scars are the most durable consequence of non-suicidal self-injury (NSSI), but how patients appraise them, and how appraisal dimensions combine within individuals, remains unclear. In this cross-sectional study, 234 adults with a history of NSSI and at least one scar, recruited consecutively in Ryazan and Moscow, Russia, completed the Russian NSSI Scar Cognition Scale (Hopelessness, Shame, Inner Strength) and measures of suicidal ideation, social anxiety, rumination, body-image dysphoria and NSSI functions. Profiles were derived by cluster analysis and tested for stability. Three stable profiles emerged: low-salience (55.1%), negative-dominant (28.2%) and strength-dominant (16.7%). The negative-dominant profile showed the highest suicidal ideation, social anxiety, rumination, body-image dysphoria and self-punishment; the strength-dominant profile showed suicidal ideation and social anxiety similar to the low-salience profile, intermediate rumination and body-image dysphoria, and the strongest endorsement of NSSI functions. These differences persisted after covariate adjustment. Scar appraisal explained a further 7.9% of variance in suicidal ideation beyond demographics, self-injury status and general distress; scar visibility explained none. In behavioural remission, the negative-dominant profile reported higher suicidal ideation than the other profiles. Scar appraisal distinguishes subgroups with different clinical correlates. Longitudinal studies are needed to establish whether these profiles predict outcomes or can guide treatment.

## 1. Introduction

Non-suicidal self-injury (NSSI) is the deliberate, self-inflicted destruction of body tissue without suicidal intent. Its prevalence differs markedly by age. In a meta-analysis of non-clinical samples, pooled lifetime prevalence was 17.2% among adolescents, 13.4% among young adults and 5.5% among adults aged over 25 years (Swannell et al., 2014). Individual surveys report 17–36% in adolescent and student populations, and 40–61% in clinical samples (Darin & Zaitseva, 2023; Kibitov et al., 2025; Kiekens et al., 2023; M. S. Zinchuk et al., 2019). Self-injury is thus most common in adolescence and early adulthood, the period in which most NSSI scars are also acquired. The clinical importance of NSSI extends beyond the episodes themselves, since a history of self-injurious thoughts and behaviours is among the most robust prospective predictors of later suicidal ideation and suicide attempts (Ribeiro et al., 2016). One mechanism proposed for this transition is acquired capability for suicide. Repeated self-injury is thought to habituate a person to physical pain and to the fear of bodily harm, which lowers one of the barriers between suicidal thoughts and suicidal acts (Hepp et al., 2026).

Scars are the objectified form of the negative affect that accompanies self-injury. The emotional experience of the act is inscribed on the body and becomes a physical object that the person can see and return to. Unlike the behaviour, which may remit, scars are permanent, and they signal that self-injury has taken place both to other people and to the person themselves. Their clinical significance follows from this double visibility. A permanent marker that others can read exposes the person to social judgement and to the anticipation of such judgement (Dyer et al., 2013; Guo et al., 2025; Lewis et al., 2025; Victor et al., 2025), while at the same time keeping the memory of the distress that produced it continuously available. Both pathways have been associated with poorer body image, psychosocial functioning and quality of life, and with slower recovery (Linington, 2024). In a cross-sectional study of young adults, Burke et al. (2016) found that the presence and number of NSSI scars were associated with current suicidal ideation and with a history of suicide attempts over and above NSSI frequency, method and medical severity, and that the association with suicidal ideation was stronger among individuals with higher levels of brooding. Qualitative studies have repeatedly documented that scars function as a standing biographical statement to which the patient has to keep positioning themselves (Bachtelle & Pepper, 2015; Chandler, 2014; Lewis & Mehrabkhani, 2016). As one adolescent in psychotherapy expressed it, “I can’t escape my scars, even if I do get better” (Kristen et al., 2024).

At the same time, the observable morphology of self-harm scars may be an insufficient guide to clinical risk on its own, that is, to the risk of suicide and of continued or escalating self-injury. In a cross-sectional study of 115 adolescents and young people with arm scars following self-harm, total scar scores were associated with self-injury frequency and several clinical characteristics, but in ROC analysis the total scar score did not significantly predict suicide risk (AUC = 0.562; *p* = 0.249) (Shi et al., 2026). Scar morphology is therefore better treated as contextual clinical information than as a direct proxy for psychopathological severity or suicide risk. These findings also point to the importance of how scars are appraised and emotionally experienced by the individual. Within cognitive-emotional accounts of NSSI, appraisals of the self, of the behaviour and of its perceived consequences, including enduring consequences such as scars, may shape whether self-injury is maintained, escalated or relinquished (Dawkins et al., 2019).

Cognitive appraisal of scars was operationalised by Burke et al. (2017) in the NSSI Scar Cognition Scale (NSSI-SCS), which distinguished reminder, social, weak, suicide and positive cognitions, and was extended by the same group to scar concealment from the self and from others, which was associated with anxiety, depression and NSSI urges even after NSSI severity had been accounted for (Burke et al., 2020). The instrument has since been taken up beyond the English-speaking context and validated in several countries, which makes scar-related cognitions comparable across languages and cultures. A Korean team subsequently developed and validated the multidimensional Korean NSSI Scar Scale (K-NSSI-ScarS) in 333 adults, replicating a five-factor structure of scar cognition (social, positive, weak, reminder and suicide factors) and reporting that the suicide factor correlated moderately with acquired capability for suicide (r = 0.34) and that all four negative factors, but not the positive one, correlated with anxiety, depression and scar concealment (Myung et al., 2024). The Russian-language validation of the NSSI-SCS in a clinical sample yielded a more parsimonious three-factor solution (Hopelessness, Shame and Inner Strength) in which the negative dimensions correlated with suicidality, social anxiety, rumination and body-image dysphoria, whereas Inner Strength was statistically independent of general distress but related to several functions of self-injury (Grigoreva et al., 2025).

Psychometric instruments are indispensable for the study of scar-related cognitions because they translate subjective experience into measurable and clinically interpretable dimensions. They permit the identification of relevant domains, comparison across individuals, and the examination of associations with NSSI severity, suicidality, and recovery-related outcomes. However, dimensional scores should not be interpreted in isolation. Variable-centred analyses identify the average contribution of separate appraisal dimensions, whereas person-centred approaches examine how these dimensions co-occur within individuals. Thus, two people with comparable scores on a single dimension, such as hopelessness, may nevertheless present with different overall patterns of shame, perceived social judgment, suicide-related cognition, self-acceptance, or positive meaning-making. Such differences may be clinically consequential. Person-centred studies of NSSI have so far addressed three questions: which combinations of methods and functions distinguish subgroups of people who self-injure (Klonsky & Olino, 2008), how indicators of recovery cluster among people with lived experience of NSSI (Hasking & Lewis, 2024), and how the anatomical location and visibility of injuries co-occur with their functions, expectancies and behavioural severity (Hon et al., 2026). Research on scars, by contrast, has remained largely variable-centred. Mostly cross-sectional studies have related scar presence to suicidal ideation and attempts (Burke et al., 2016), scar concealment to anxiety, depression and NSSI urges (Burke et al., 2020), and negative scar cognitions to anxiety, depression, shame, rumination and body-image dysphoria (Grigoreva et al., 2025; Myung et al., 2024). Associations of scar appraisal itself with suicide-related outcomes are fewer and less direct: with acquired capability for suicide in a Korean sample (Myung et al., 2024) and with suicidal ideation in a Russian clinical sample (Grigoreva et al., 2025). Perceptions of scarring have entered one person-centred model, but only as one of several indicators of recovery (Hasking & Lewis, 2024), and a recent systematic review of data-driven NSSI subgroups identified no study in which subgroups were derived from scar-related cognitions (Kruzan et al., 2025). To our knowledge, no study has yet examined how the dimensions of scar appraisal combine within the same person. The gap addressed here is therefore not whether scar appraisal is associated with distress, which has been reported in several cross-sectional studies, but whether it organises into distinct person-level configurations with different clinical correlates. Applying person-centred methods to validated scar-cognition measures may therefore complement psychometric assessment rather than replace it, by identifying clinically meaningful configurations of experience.

Recovery from NSSI has often been operationalised primarily as behavioural cessation. Although abstinence is an important outcome, it may not fully capture recovery-related change in emotional functioning, self-concept, coping resources, urges to self-injure, or the psychological impact of the enduring consequences of NSSI (Hasking & Lewis, 2024; Hudson et al., 2024). Scars are one such enduring consequence. For some individuals, scars may remain associated with shame, concealment, perceived social judgment, self-criticism, or reminders of prior distress even after self-injury has ceased. Accordingly, the absence of recent NSSI should not be assumed to indicate the absence of clinically relevant scar-related distress. This possibility highlights the value of assessing scar appraisals alongside behavioural indicators when characterising recovery from NSSI.

Scar appraisal can also be situated within cognitive models of trauma. In the cognitive model of posttraumatic stress disorder, persistent distress is maintained not by the event alone but by negative appraisals of the event and of its sequelae, which produce a sense of current threat, shame and a changed view of the self (Ehlers & Clark, 2000; Foa et al., 1999). Rumination about the event and its consequences is one of the cognitive strategies that keep such appraisals active (Michael et al., 2007). Evidence from the acute phase after trauma points in the same direction. Acute stress reactions are common in the first weeks after a traumatic event, and the diagnosis of acute stress disorder identifies only a minority of those who later develop chronic posttraumatic stress disorder (Bryant, 2011a). In prospective studies of assault and motor vehicle accident survivors, cognitive variables assessed within the first weeks, including negative appraisals of the event and its consequences, mental defeat and rumination, predicted later posttraumatic stress more strongly than initial symptom severity or established risk factors (Ehring et al., 2008; Kleim et al., 2007). Whether acute distress resolves or becomes chronic thus appears to depend less on the intensity of the initial reaction than on how the event and its physical and social consequences come to be appraised and ruminated upon. Although self-injury differs from externally inflicted trauma in being self-initiated, it is most often undertaken to regulate acute negative affect (Klonsky, 2007; Nock, 2009), which may itself arise as a heightened, over-sensitive reaction to stress: adolescents who self-injure show greater physiological reactivity and poorer distress tolerance under stress than those who do not (Nock & Mendes, 2008). Each episode is thus an acute bodily event embedded in an acute stress reaction that leaves a permanent physical sequela, and the appraisals formed around the wound and the resulting scar may likewise either fade or consolidate over time. Suicide-related cognitions have been shown to contribute to suicide risk alongside depression among people reporting posttraumatic stress symptoms (Rugo et al., 2020), and fluid vulnerability theory describes such cognitions as dynamic components of suicide risk that fluctuate over time rather than as fixed traits (Rugo-Cook et al., 2021). Scars can be understood in this framework as a permanent and potentially visible sequela of past distress to which the same appraisal processes apply: whether they are read as evidence of damage and entrapment or as evidence of survival may shape their emotional impact, and rumination may be one route through which a negative reading is maintained. Because the meaning of self-injury, and of its visible marks, is shaped by cultural norms concerning the body, the family and the social self (X. Chen et al., 2021; Gholamrezaei et al., 2017; Guo et al., 2025), appraisal patterns identified in one cultural setting cannot be assumed to hold in others and need to be examined across cultural and linguistic contexts. The same holds for trauma-related cognitions themselves: intrusive memories and negative alterations in cognition and mood were the posttraumatic symptom clusters with the highest predictive value for PTSD, and their predictive value depended on contextual factors (Figueiredo & Ndiaye, 2025).

The present study therefore had four aims: (1) to identify person-level profiles of cognitive appraisal of NSSI scars in adults with a history of self-injury; (2) to characterise these profiles on suicidal ideation, social anxiety, rumination, body-image dysphoria and the functions of self-injury; (3) to test whether scar appraisal explains variance in suicidal ideation over and above demographic characteristics, current self-injury status, scar visibility and general psychological distress; and (4) to test whether appraisal profiles differentiate suicidal ideation among participants who have already achieved behavioural remission. The overarching purpose is to examine whether cognitive appraisal of scars carries clinically relevant information that would justify its inclusion as a routine assessment domain and its evaluation, in future longitudinal and intervention studies, as a potential focus of psychotherapeutic work in this vulnerable group.

## 2. Materials and Methods

### 2.1. Study Design

This was a cross-sectional observational study reported in accordance with the STROBE statement (von Elm et al., 2007). It is a secondary analysis of the dataset collected in the parent project on the consequences of non-suicidal self-injury, a subsample of which was used for the psychometric validation of the Russian-language NSSI-SCS reported elsewhere (Grigoreva et al., 2025). The present analysis addresses a different question—the person-level configuration of scar appraisal and its clinical correlates—and uses a differently defined analytic sample; no result reported here is reproduced from the validation study. The parent project was approved by the Local Ethics Committee of Ryazan State Medical University before recruitment began (protocol No. 1, 12 September 2023), and the present secondary analysis of the anonymised data was approved by the same committee (protocol No. 3, 6 May 2025).

### 2.2. Setting and Participants

Participants were recruited by consecutive sampling between November 2023 and January 2025 from the inpatient and outpatient departments of the N.N. Bazhenov Regional Clinical Psychiatric Hospital (Ryazan) and the outpatient neuropsychiatric clinic “Neurocenter” (Moscow).

Inclusion criteria for the present analysis were: (1) age 18 years or older; (2) a self-reported history of deliberate self-injury on at least five days within a single 12-month period, a threshold corresponding to the frequency criterion for NSSI disorder in DSM-5 (American Psychiatric Association, 2013), where that period could be either the 12 months preceding assessment or any earlier year (individuals who had never self-injured on five or more days within one year were not included); (3) at least one self-reported NSSI scar; and (4) a complete NSSI-SCS protocol (that is, a fully completed NSSI-SCS form with no missing items). Exclusion criteria applied at recruitment were a documented history of organic mental disorder, substance use disorder, schizophrenia spectrum disorder or intellectual disability, and a current psychotic episode, acute intoxication, catatonia or clinically significant cognitive impairment, as established by clinical examination and review of medical records.

Of 336 individuals assessed, 241 met the self-injury and scar criteria, and 234 of these were aged 18 years or older and formed the analytic sample. No data were missing on the NSSI-SCS; the Beck Scale for Suicide Ideation was incomplete for four participants (1.7%), who were excluded listwise from the regression analyses only.

### 2.3. Measures

Cognitive appraisal of scars. The Russian-language NSSI Scar Cognition Scale (Burke et al., 2017; Grigoreva et al., 2025) was administered in its validated 21-item three-factor form. Items are rated from 1 (“not at all”) to 5 (“extremely”) and yield three subscales: Hopelessness (9 items; scar-related cognitions of entrapment, weakness, hopelessness and suicide; range 9–45), Shame (6 items; anticipated social judgement, embarrassment and unattractiveness; range 6–30) and Inner Strength (6 items; scars as evidence of endurance, pride and resilience; range 6–30). Internal consistency in the present sample was good to excellent: Hopelessness α = 0.883, ω = 0.889; Shame α = 0.871, ω = 0.874; Inner Strength α = 0.828, ω = 0.839.

Suicidal ideation. The Beck Scale for Suicide Ideation (BSSI) (Beck et al., 1979) quantifies the intensity of current suicidal ideation and intent.

Social anxiety. The Liebowitz Social Anxiety Scale (LSAS), Russian version (Grigorieva & Enikolopov, 2016), measures fear and avoidance across 24 social and performance situations.

Rumination. The Ruminative Responses Scale (RRS), Russian version (Pugovkina et al., 2021), assesses a passive, repetitive focus on negative affect and its causes.

Body-image dysphoria. The Situational Inventory of Body-Image Dysphoria (SIBID), Russian adaptation (Baranskaya et al., 2008), measures context-specific body-image distress.

Functions of self-injury. The Inventory of Statements about Self-Injury (ISAS-II) (Klonsky & Glenn, 2009; M. Zinchuk et al., 2023) assesses 13 functions of NSSI, grouped into an intrapersonal domain (affect regulation, self-punishment, anti-dissociation, anti-suicide) and an interpersonal domain (interpersonal boundaries, self-care, sensation seeking, peer bonding, interpersonal influence, toughness, revenge, autonomy, marking distress).

Self-injury status and scar location. Two purpose-designed screening items were used. (a) Self-injury status was recorded with a single forced-choice item with three response options: never self-injured; self-injured on at least five days during the past 12 months (“current”); or previously self-injured on at least five days within a year but not during the past six months or longer (“remitted”). Participants were classified according to the single option they selected; the date of the most recent episode was not recorded. The six-month threshold was chosen as a pragmatic marker of sustained cessation, intended to separate it from the brief interruptions that are common in the course of NSSI while retaining an adequately sized remitted subgroup; it is not an empirically established cut-off. Because the two options overlap for individuals whose most recent episode occurred between six and twelve months before assessment and who had self-injured on at least five days during that year, such individuals could have endorsed either option, and the extent of this overlap cannot be determined from the available data (see Section 4.2). (b) A second item recorded whether scars were located on exposed body areas visible to others, on concealed areas, or both. For analysis, scar location was dichotomised into any visible scarring versus concealed scarring only.

### 2.4. Statistical Analysis

The distribution of all continuous variables departed significantly from normality (Shapiro–Wilk, *p* < 0.05), and non-parametric tests were therefore used for all unadjusted group comparisons. The hierarchical regression and the covariate-adjusted profile comparisons described below were nevertheless estimated by ordinary least squares, because the distributional assumption of these models concerns the residuals rather than the raw variables; their assumptions were checked explicitly, their inference was based on heteroscedasticity-consistent standard errors and bootstrap confidence intervals, and their robustness was examined in sensitivity analyses.

Profiles of scar appraisal were derived from the three NSSI-SCS subscale scores after z-standardisation. The number of clusters was determined by triangulating three criteria: the silhouette coefficient for Ward’s hierarchical solutions and for k-means solutions with k = 2–6; the Bayesian information criterion (BIC) and classification entropy of Gaussian finite-mixture models (diagonal covariance, regularisation 0.10) over the same range; and the substantive interpretability and minimum size of the resulting groups. The final solution was estimated by k-means with 500 random initialisations and a fixed random seed. The stability of the retained solution was examined in three ways: cluster-wise bootstrap stability, that is, the mean Jaccard similarity between each original cluster and its best-matching cluster across 1000 bootstrap resamples, with values of 0.75 or above indicating a stable and 0.85 or above a highly stable cluster (Hennig, 2007); split-half replication across 500 random splits, in which the solution derived in one half was used to classify the other half and compared with that half’s own solution by the adjusted Rand index (ARI); and prediction strength for k = 2–5 (Tibshirani & Walther, 2005). Agreement of the k-means solution with the three-cluster Ward and Gaussian-mixture solutions, and its correspondence with the four-cluster k-means solution, were also examined.

Profiles were compared using the Kruskal–Wallis test with epsilon-squared (ε^2^) as the effect size, followed by pairwise Mann–Whitney U tests with Holm correction applied within each dependent variable and the effect size r = |Z|/√*N*. Categorical variables were compared with the χ^2^ test. Because the profiles differed in several sociodemographic and clinical characteristics (Table 1), each clinical outcome was additionally regressed on profile membership with adjustment for age, gender, education, occupational status, current self-injury status and scar visibility, using HC3 standard errors. These models were estimated both on raw and on rank-transformed outcome scores (a rank-based analysis of covariance), and the three pairwise profile contrasts were Holm-corrected within each outcome. The comparison of suicidal ideation across profiles within the remitted subgroup, and between remitted participants with a low-salience or a negative-dominant appraisal and participants who had self-injured in the past year, was adjusted in the same way (without self-injury status).

Incremental prediction of suicidal ideation was tested by hierarchical ordinary least squares regression on the BSSI total score: Step 1 entered age, gender, current self-injury status and scar visibility; Step 2 added rumination, body-image dysphoria and social anxiety; Step 3 added the three NSSI-SCS subscales. Heteroscedasticity-consistent (HC3) standard errors were used, 95% confidence intervals for the key coefficients were obtained by bootstrap resampling (5000 replications), and multicollinearity was assessed by variance inflation factors. Regression assumptions were examined through the distribution of residuals (skewness, kurtosis and the Shapiro–Wilk test), the Breusch–Pagan test for heteroscedasticity, the RESET test for functional form, and Cook’s distance and externally studentised residuals for influential observations. As sensitivity analyses, the final model was re-estimated on rank-transformed BSSI scores, by Huber robust regression, and after exclusion of influential cases (Cook’s distance >4/*n*). Throughout, the term “predictor” is used in its statistical sense and does not imply temporal precedence or causation.

Two-tailed *p*-values below 0.05 were considered statistically significant. Analyses were performed in Python 3.9 (pandas 2.3, SciPy 1.13, statsmodels 0.14, scikit-learn 1.6). The additional analyses conducted in response to peer review (cluster-stability analyses, covariate-adjusted profile comparisons, and regression diagnostics and sensitivity analyses) were carried out with the generative AI assistant Claude (Anthropic; Claude Opus 5.5), which wrote and executed the Python code for these analyses; the authors specified the analyses, checked the code and verified the results.

## 3. Results

### 3.1. Sample Characteristics

The analytic sample comprised 234 adults (mean age 24.34 years, SD 6.92, range 18–69). Most were women (174; 74.4%). Self-injury was current in 85 participants (36.3%) and remitted for more than six months in 149 (63.7%). Scars were located on exposed areas only in 119 participants (50.9%), on concealed areas only in 71 (30.3%) and in both locations in 44 (18.8%); 163 participants (69.7%) therefore had at least some visible scarring. Full sociodemographic and clinical characteristics, overall and by appraisal profile, are presented in Table 1.

### 3.2. Derivation of Appraisal Profiles

Silhouette coefficients for the k-means solutions were 0.403 (k = 2), 0.416 (k = 3), 0.348 (k = 4) and 0.349 (k = 5); Ward’s hierarchical solutions yielded comparable values (0.408 for k = 2 and 0.389 for k = 3). The BIC of Gaussian mixture models fell steeply from 2025.0 (k = 1) to 1859.1 (k = 2) and 1805.0 (k = 3), with only a negligible further decrease at k = 4 (1800.5) before rising again; classification entropy was 0.71 at k = 3. The three-cluster solution was therefore retained: it had the highest silhouette coefficient, although only marginally above the two-cluster solution, sat at the elbow of the BIC curve, produced no group smaller than 16% of the sample, and was the most clinically interpretable.

The retained solution was stable under resampling (Supplementary Table S1). Mean bootstrap Jaccard similarities were 0.94 for Profile 1, 0.97 for Profile 2 and 0.86 for Profile 3, all within the range indicating a highly stable cluster (5th percentiles 0.85, 0.80 and 0.67, respectively). In split-half replication, classification of one half by the centroids of the other agreed closely with that half’s own solution (median ARI = 0.82, interquartile range 0.76–0.88) and with the full-sample profiles (median ARI = 0.91). Prediction strength was 0.87 for k = 2, 0.71 for k = 3, 0.50 for k = 4 and 0.40 for k = 5: solutions with more than three clusters did not replicate across random halves, whereas the three-cluster solution replicated moderately rather than strongly. The four-cluster solution did not introduce a qualitatively new configuration. The strength-dominant profile was retained almost unchanged (38 of its 39 members), and the additional cluster arose from dividing the negative-dominant profile into a more severe group (*n* = 32) and an intermediate group (*n* = 59) that also absorbed 24 members of the low-salience profile, thus adding a gradation of severity rather than a distinct pattern. Agreement of the k-means solution with the three-cluster Ward (ARI = 0.66) and Gaussian-mixture (ARI = 0.47) solutions was moderate, indicating that the exact boundaries between profiles depend on the algorithm used. The profiles are therefore best regarded as a parsimonious and reasonably stable description of this dataset rather than as fixed categories.

**Figure 1:**
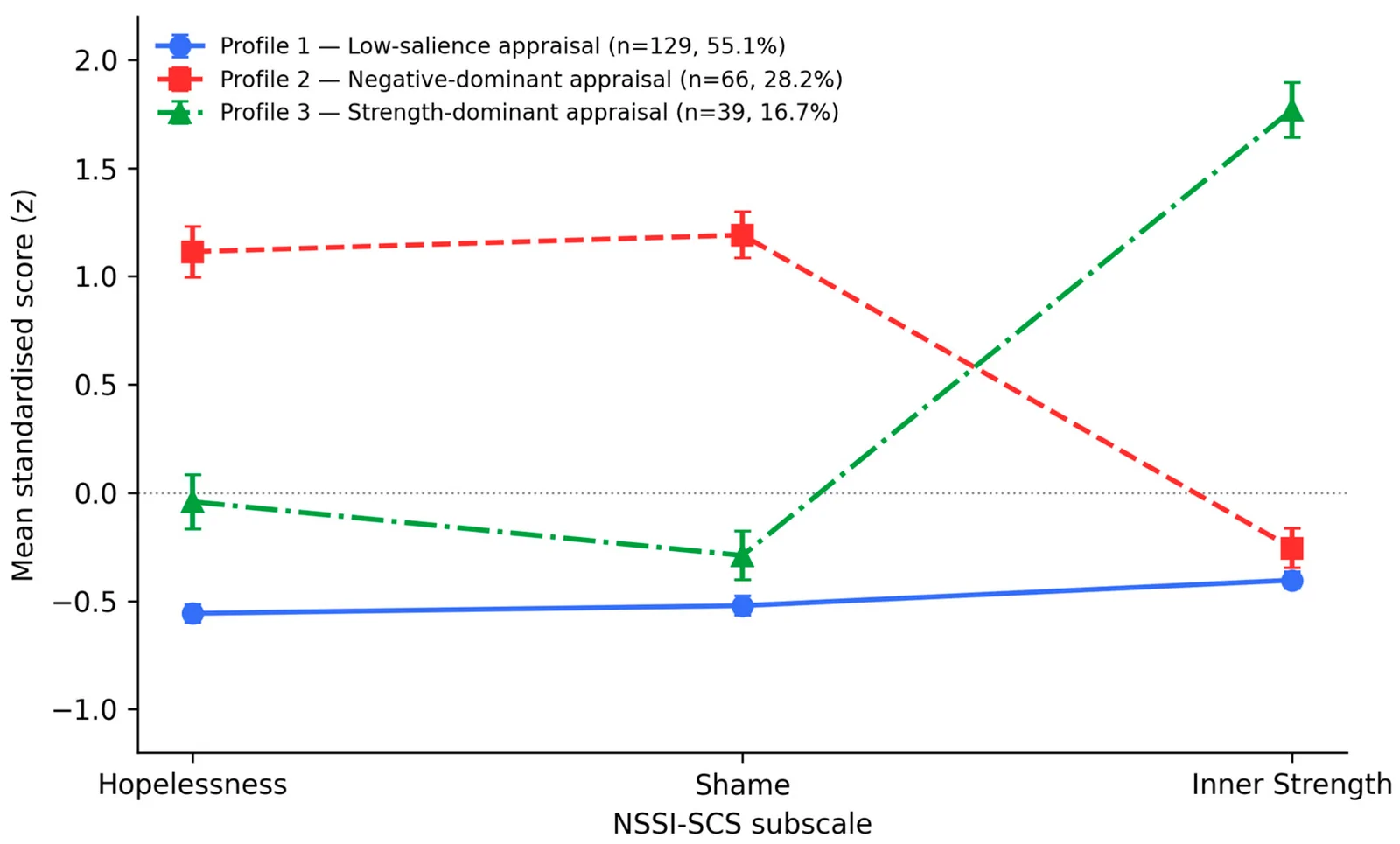
Standardised mean NSSI Scar Cognition Scale (NSSI-SCS) subscale scores of the three profiles of scar appraisal (*N* = 234). Values are group means of z-standardised subscale scores; error bars are standard errors of the mean. The dotted line marks the sample mean.

The three profiles are shown in Figure 1 and quantified in Table 2. Profile 1 (*n* = 129; 55.1%), labelled low-salience appraisal, was defined by below-average scores on all three subscales. For these participants scars were not a psychologically loaded object. Profile 2 (*n* = 66; 28.2%), labelled negative-dominant appraisal, combined markedly elevated Hopelessness and Shame with an absent sense of inner strength. Profile 3 (*n* = 39; 16.7%), labelled strength-dominant appraisal, was defined by a pronounced elevation on Inner Strength (z = +1.77) alongside average Hopelessness and below-average Shame. Between-profile differences on all three subscales were large (Hopelessness H = 108.87, ε^2^ = 0.463; Shame H = 110.01, ε^2^ = 0.468; Inner Strength H = 95.37, ε^2^ = 0.404; all *p* < 0.001).

### 3.3. Clinical Characteristics of the Profiles

Profile 2 was the clinically most severe group on every general measure of distress. Compared with Profile 1, participants with a negative-dominant appraisal reported higher suicidal ideation (Holm-corrected *p* < 0.001, r = 0.28), social anxiety (*p* < 0.001, r = 0.37), body-image dysphoria (*p* < 0.001, r = 0.41) and rumination (*p* < 0.001, r = 0.48), and endorsed self-punishment as a function of self-injury far more strongly (*p* < 0.001, r = 0.36).

Profile 3 presented a different and less obvious picture. On suicidal ideation, participants with a strength-dominant appraisal did not differ from the low-salience profile (*p* = 0.627, r = 0.04) and scored significantly lower than the negative-dominant profile (*p* = 0.025, r = 0.25); the same pattern held for social anxiety. On the functions of self-injury, however, Profile 3 was the most strongly endorsing group of all three. It exceeded both other profiles on sensation seeking (vs. Profile 1 *p* < 0.001, r = 0.40; vs. Profile 2 *p* < 0.001, r = 0.40) and scored highest on the interpersonal domain overall (vs. Profile 1 *p* < 0.001, r = 0.42; vs. Profile 2 *p* = 0.013, r = 0.24), on toughness (vs. Profile 1 *p* < 0.001, r = 0.28), on autonomy (vs. Profile 1 *p* = 0.010) and on marking distress (vs. Profile 1 *p* < 0.001, r = 0.30). A positive appraisal of scars thus co-occurred with a stronger investment of self-injury in identity, in self-testing and in communication with others, and not with a lower level of distress.

Covariate-adjusted comparisons (Supplementary Table S2). After adjustment for age, gender, education, occupational status, current self-injury status and scar visibility, profile membership remained significantly associated with every clinical outcome and every function of self-injury examined (all *p* ≤ 0.008), with concordant results for raw and rank-transformed scores. The differences between the negative-dominant and low-salience profiles in suicidal ideation, social anxiety, body-image dysphoria, rumination and self-punishment all remained significant (Holm-corrected *p* ≤ 0.006), as did the higher endorsement of sensation seeking, interpersonal functions, toughness and marking distress in the strength-dominant compared with the low-salience profile (Holm-corrected *p* ≤ 0.003) and its higher sensation seeking and interpersonal functions compared with the negative-dominant profile (Holm-corrected *p* ≤ 0.043). The lower social anxiety and rumination of the strength-dominant compared with the negative-dominant profile also remained significant (Holm-corrected *p* ≤ 0.009). One contrast was attenuated: the lower suicidal ideation of the strength-dominant compared with the negative-dominant profile was no longer statistically significant after adjustment (B = −2.68, *p* = 0.062; Holm-corrected *p* = 0.124).

Item-level endorsement made the divergence between profiles concrete (Table 3). Among participants with a negative-dominant appraisal, one in three (33.3%) endorsed at least moderately the statement “My scars make me feel like I want to kill myself”, against 0.8% in the low-salience profile; 69.7% endorsed “My scars make me feel like I could kill myself if I wanted to”, the item most closely aligned with acquired capability; and 83.3% reported at least moderate embarrassment in front of other people.

### 3.4. Scar Appraisal and Suicidal Ideation

At the zero-order level, suicidal ideation correlated moderately with Hopelessness (Spearman’s ρ = 0.418, *p* < 0.001), weakly with Shame (ρ = 0.135, *p* = 0.041) and not at all with Inner Strength (ρ = −0.064, *p* = 0.336).

The hierarchical regression is reported in Table 4. Demographic and behavioural variables explained 13.3% of the variance in suicidal ideation (F(4, 225) = 8.64, *p* < 0.001), with current self-injury the dominant predictor (β = 0.283). Adding rumination, body-image dysphoria and social anxiety raised the explained variance to 26.2%. Adding the three scar-appraisal subscales in Step 3 produced a further significant increment of 7.9% (ΔR^2^ = 0.079; F(3, 219) = 8.80, *p* < 0.001), bringing the total explained variance to 34.1% (adjusted R^2^ = 0.311). Hopelessness was the strongest single predictor in the final model (β = 0.374, *p* < 0.001; bootstrap 95% CI for B 0.221 to 0.490), exceeding both current self-injury status (β = 0.177) and rumination (β = 0.246). Scar visibility was not a significant predictor at any step. All variance inflation factors were below 2.6. Regression diagnostics showed mild positive skew of the residuals (skewness 0.48, excess kurtosis 0.61; Shapiro–Wilk *p* = 0.010), heteroscedasticity (Breusch–Pagan *p* = 0.014), which was addressed by the HC3 standard errors, no evidence of misspecified functional form (RESET *p* = 0.944) and no single dominant influential case (maximum Cook’s distance 0.12). The increment of scar appraisal and the coefficients for Hopelessness and Shame were robust to rank transformation of the outcome (ΔR^2^ = 0.085; Hopelessness β = 0.386, Shame β = −0.253, both *p* < 0.001), to Huber robust regression (both *p* < 0.001) and to exclusion of 12 influential cases (ΔR^2^ = 0.134; Hopelessness β = 0.485, Shame β = −0.307, both *p* < 0.001; Supplementary Table S3).

Shame carried a significant negative coefficient in the final model (β = −0.244, *p* < 0.001; bootstrap 95% CI −0.466 to −0.132) despite a positive zero-order correlation with suicidal ideation. This is a suppression pattern. Taken on its own, scar-related shame goes together with slightly higher suicidal ideation, but almost all of that association is carried by what shame shares with hopelessness about scars and with general social anxiety. Once those two are held constant, the part of shame that is specific to it is associated with lower, not higher, suicidal ideation. For two patients who are equally hopeless about their scars and equally socially anxious, the one who is more ashamed of them reports somewhat less suicidal ideation. Substantively, shame about scars presupposes a continued investment in how one is seen by others, which is not the same psychological position as the entrapment and futility captured by Hopelessness. The finding should be treated as hypothesis-generating and requires replication, because a coefficient that reverses sign relative to its zero-order correlation is by definition conditional on the particular set of covariates entered into the model: it need not survive a different model specification, a different sample, or a measure of shame that is not restricted to scars.

### 3.5. Scar Appraisal After Behavioural Remission

Negative appraisal was, as expected, more pronounced in participants who had self-injured in the past year than in those in remission (Hopelessness 20.91 ± 8.60 vs. 16.66 ± 7.34, *p* < 0.001, r = 0.27; Shame 13.74 ± 6.31 vs. 11.57 ± 5.73, *p* = 0.005, r = 0.18). The effect sizes were small, however, and negative appraisal did not disappear with behavioural cessation: 21.5% of the 149 participants in remission for more than six months fell into the negative-dominant profile.

These participants reported comparatively elevated suicidal ideation. Within the remitted group, BSSI scores differed significantly by profile (H = 8.43, *p* = 0.015): 18.13 ± 7.28 in the negative-dominant profile versus 13.72 ± 7.70 in the low-salience profile (*p* = 0.010, r = 0.24) and 13.07 ± 6.27 in the strength-dominant profile (*p* = 0.007, r = 0.35). The suicidal ideation of remitted participants with a negative-dominant appraisal did not differ significantly from that of participants who had self-injured in the past year (18.13 ± 7.28 vs. 19.48 ± 6.55; *p* = 0.380, r = 0.08), whereas remitted participants with a low-salience appraisal scored substantially lower than participants who had self-injured in the past year (*p* < 0.001, r = 0.37). These results held after adjustment for age, gender, education, occupational status and scar visibility: within the remitted subgroup, profile remained associated with suicidal ideation (F(2, 133) = 4.02, *p* = 0.020); remitted participants with a negative-dominant appraisal did not differ from participants who had self-injured in the past year (adjusted B = −1.30, *p* = 0.392), whereas remitted participants with a low-salience appraisal scored lower (adjusted B = −5.38, *p* < 0.001). A non-significant difference does not establish equivalence, and in this cross-sectional sample it cannot be determined whether appraisal changed over the course of remission; the observation is that behavioural remission was accompanied by lower suicidal ideation only among participants who did not currently hold a negative-dominant appraisal.

Scar visibility, by contrast, showed the dissociation reported previously (Grigoreva et al., 2025): participants with any visible scarring scored higher on Inner Strength than those whose scars were concealed (10.18 ± 4.89 vs. 8.37 ± 3.78; *p* < 0.001, r = 0.23) but did not differ on Hopelessness (*p* = 0.614) or Shame (*p* = 0.935).

## 4. Discussion

This study asked whether the way a person appraises their self-injury scars carries clinically relevant information. Three findings suggest that it does, although, given the cross-sectional design, they describe associations and cannot show that appraisal should determine the choice of treatment.

First, scar appraisal does not behave as a single dimension of severity; it organises into qualitatively distinct configurations. Almost half of this clinical sample (44.9%) fell into a profile in which scars were psychologically salient, and the two salient profiles differed not only from the low-salience group but also from each other on several clinical characteristics. Comparable heterogeneity has already been described within self-injury itself. Person-centred studies have identified distinct subgroups in how people recover from NSSI (Hasking & Lewis, 2024) and in how the location and visibility of injuries combine with their functions and with cognitions about them (Hon et al., 2026). The present results show that such heterogeneity is also present in how people relate to their scars, that is, in the consequence of self-injury that remains once the behaviour has stopped.

Second, the clinical meaning of the two salient profiles is different, and each suggests different clinical priorities. The negative-dominant profile (28.2%) is the configuration associated with the greatest current distress: elevated suicidal ideation, social anxiety, rumination and body-image dysphoria, with self-punishment as the leading function of self-injury. One in three of these patients endorsed scar-triggered suicidal wishes at least moderately, and seven in ten endorsed the sense of being able to kill themselves if they chose to, the cognition most directly aligned with acquired capability, which the Korean validation found to correlate with the suicide factor of scar cognition (Myung et al., 2024) and which pain-habituation studies place at the centre of the transition from self-injury to suicidality (Hepp et al., 2026). For this group the appraisal itself may be a relevant therapeutic focus, above all shame, self-criticism and the sense of entrapment. In this profile the scars appear to be experienced as an active source of present distress and not only as a record of past behaviour; they are appraised as evidence of personal failure and as grounds for the belief that nothing can change. In our data these appraisals were associated with suicidal ideation over and above current self-injury status and general distress. This configuration parallels the role that negative appraisals of a trauma and of its sequelae, together with rumination about them, play in maintaining posttraumatic distress (Ehlers & Clark, 2000; Michael et al., 2007), a parallel reinforced by the markedly elevated rumination in this profile. After acute trauma, early rumination and mental defeat are among the strongest predictors of chronic posttraumatic stress (Kleim et al., 2007). By analogy, hopelessness about scars combined with elevated rumination in this profile may reflect appraisals that became fixed rather than resolved after the acute phase of self-injury. This hypothesis requires prospective testing. If the association reflects a maintaining process, which only longitudinal and intervention studies can establish, an intervention confined to emotion regulation or to relapse prevention might leave it unaddressed. Compassion-focused and self-compassion-based interventions have reduced NSSI, shame and self-criticism in controlled and pilot trials (Gholizadeh & Pourmohammad, 2026; Hu et al., 2026), and the broader evidence base for psychosocial interventions in self-harm supports structured, cognitively oriented work over generic supportive contact (Tan & Zhang, 2026; Witt et al., 2021a, 2021b). Clinically, this is also the group in which requests for dermatological or surgical scar treatment (Edriss et al., 2022; Ho et al., 2018) should be explored since the shame that motivates the request is itself a treatment target and the way clinicians talk about scars carries ethical weight (Islam et al., 2026).

In cognitive models of posttraumatic stress, trauma memories are triggered by external sensory cues such as places, sounds or smells (Ehlers & Clark, 2000). A scar differs from such cues. It is permanent, it is located on the person’s own body, and the person produced it. It cannot be avoided by staying away from a particular place or situation, so the remaining options are to conceal it, to avoid looking at it or to change its meaning. Self-injury is usually repetitive, and each episode may leave its own scar. Over time the body becomes an autobiographical archive of emotional crises. Such an archive may keep these crises central to the person’s life story. In the negative-dominant profile the scars appear to be read as a record of defeat. In the strength-dominant profile they are read as a record of survival, which may keep self-injury close to identity. In the low-salience profile they seem to have lost much of their narrative significance.

The strength-dominant profile (16.7%) is the least expected result. On suicidal ideation and social anxiety these participants resembled the low-salience group, and a clinician reading only symptom scores might classify them as low priority. On rumination and body-image dysphoria they occupied an intermediate position, and after covariate adjustment their suicidal ideation no longer differed significantly from that of the negative-dominant profile either. Yet they endorsed the functions of self-injury more strongly than any other profile: sensation seeking, toughness, autonomy, marking distress, and the interpersonal domain as a whole. In these data, appraising one’s scars as evidence of endurance was associated less with markers of recovery than with a stronger endorsement of functions that link self-injury to identity and to the way distress is communicated. This is consistent with the finding in the Russian validation study that Inner Strength was unrelated to distress but related to several NSSI functions (Grigoreva et al., 2025), with the Korean observation that the positive factor behaves differently from every other scar-cognition factor (Myung et al., 2024), and with qualitative accounts of scars as narrative resources for identity (Chandler, 2014; Kendall et al., 2021; Lewis & Mehrabkhani, 2016). It also gives reason for caution about uncritical clinical or online endorsement of the idea of “scars as badges of survival”. Building on a person’s own strengths and resources is a genuinely useful principle in preventing self-injury (Petrovic et al., 2026), but the present data suggest that when the strength is located in the scar rather than in the person, the behaviour that produced it may retain its meaning. If confirmed, this pattern would point towards meaning and identity work, separating the legitimate sense of having survived from the injury as its proof, and building alternative sources of agency, belonging and self-testing.

Third, and with the most direct implications for service delivery, scar appraisal explained variance in suicidal ideation over and above current self-injury status and general distress (ΔR^2^ = 0.079), and hopelessness about scars was more strongly associated with suicidal ideation than whether the person had self-injured in the past year; this increment was robust across the sensitivity analyses. In practical terms, in this sample what a patient made of their scars was more informative about their current suicidal ideation than whether they had self-injured in the past year. Two patients who have both stopped self-injuring may therefore differ in current suicidal ideation if one of them still reads their scars as proof that nothing can change. Scar visibility itself was not significantly associated with suicidal ideation, even though it is the property that can be observed directly. This agrees with the recent finding that the morphological characteristics of adolescent arm scarring have limited predictive utility for suicide risk (Shi et al., 2026). What carries clinical information is how the mark is interpreted rather than the mark itself. Research on traumatic physical injury points in the same direction. The psychiatric consequences of such injury extend beyond posttraumatic stress disorder to depression and anxiety disorders, and physical consequences such as pain and brain injury are closely linked with psychological symptoms and with the course of recovery (Bryant, 2011b). Self-injury scars are a lasting physical consequence of this kind, and their assessment should likewise go beyond their physical characteristics. An assessment protocol that records the presence and extent of scarring without asking the patient what it means to them therefore captures only part of the relevant information.

The remission analysis sharpens this point. Among participants who had not self-injured for more than six months, one in five still held a negative-dominant appraisal, and their suicidal ideation did not differ significantly from that of participants who had self-injured in the past year, also after covariate adjustment. Behavioural cessation was accompanied by lower suicidal ideation only among those who did not hold a negative-dominant appraisal. Recovery defined solely as the absence of the behaviour may therefore overlook a subgroup with persisting suicidal ideation. First-person accounts of recovery have long insisted that it involves more than stopping (Hudson et al., 2024), and adolescents in cognitive-behavioural therapy for depression describe scars as the part of self-injury that does not end when the behaviour does (Kristen et al., 2024). What has so far been described mainly in qualitative and first-person terms can therefore be expressed numerically and tied to a specific instrument. The state that patients and clinicians describe as “stopped but not recovered” may correspond to an identifiable score profile on the NSSI-SCS that can be counted, compared between services and followed over time; whether it predicts later suicidal behaviour remains to be tested prospectively.

The three-factor structure on which these profiles rest is itself culturally specific. The Russian solution merges the four negative dimensions of the original instrument into Hopelessness and Shame (Grigoreva et al., 2025), whereas the Korean instrument retains five (Myung et al., 2024) and the original US scale distinguishes reminder, weak, social and suicide cognitions (Burke et al., 2017). Shame emerging as a standalone dimension in the Russian sample is consistent with evidence that the social meaning of self-injury is more strongly foregrounded outside individualistic contexts (X. Chen et al., 2021; C. Chen et al., 2025; Gholamrezaei et al., 2017). Cultural context may shape not only the factor structure but also the content of appraisal: where the social consequences of self-injury for the person and the family are foregrounded, and where self-injury is more strongly stigmatised, the shame-related and social meanings of scars are likely to weigh more heavily (X. Chen et al., 2021; Guo et al., 2025). The present sample was recruited in a single country, from two specialised services, and the profiles may partly reflect the characteristics of this Russian clinical population. Profiles derived here should therefore be replicated in other cultural and linguistic contexts, including migrant and minority populations, before they are assumed to generalise across languages and health systems.

### 4.1. Implications for Practice

Three practical implications are proposed; because they are derived from cross-sectional associations, they should be tested prospectively before being adopted as standards of care. First, cognitive appraisal of scars could be assessed routinely, for example at intake and again before discharge, in patients with a history of self-injury and scarring; a validated 21-item instrument (Grigoreva et al., 2025) makes this a matter of minutes. Second, the appraisal profile may help clinicians to prioritise the focus of intervention rather than merely documenting severity: low-salience appraisal may require no scar-specific work beyond monitoring; negative-dominant appraisal suggests that shame-, self-criticism- and hopelessness-focused work, with explicit suicide risk assessment and careful handling of requests for scar removal, may be appropriate; strength-dominant appraisal suggests identity- and meaning-focused work and the substitution of the interpersonal and self-testing functions that self-injury currently serves. Whether matching interventions to appraisal profiles improves outcomes has not yet been tested. Third, behavioural remission may not be a sufficient endpoint of care while a negative appraisal of scars persists; in this sample, that combination was accompanied by a level of suicidal ideation that did not differ significantly from that of participants who had self-injured in the past year.

### 4.2. Strengths and Limitations

The findings rest on a clinically recruited sample of adequate size and on a validated culture-specific measure of the central construct. The person-centred design was triangulated across three clustering criteria, the stability of the profiles was examined by resampling, inference used heteroscedasticity-consistent standard errors and bootstrap confidence intervals, the main comparisons were re-examined with covariate adjustment, and the behavioural-remission subgroup made it possible to examine the question of recovery within the sample.

Its limitations constrain the conclusions. The design is cross-sectional, so no causal or temporal claim can be made. Negative scar appraisal may drive suicidal ideation, follow from it, or share a common cause. Cluster solutions are sample-dependent and descriptive. The three profiles are a parsimonious summary of this dataset, not established taxa: the three-cluster solution showed high bootstrap stability but only moderate prediction strength and moderate agreement across clustering algorithms, the profiles have not been validated in an independent sample, and they may partly reflect characteristics of this particular Russian clinical sample. They therefore require replication in independent and preferably longitudinal samples. The sample was predominantly female (74.4%), young and help-seeking, which limits generalisation to men, to older adults and to people not in contact with services. Participants were recruited in a single country and from two specialised psychiatric services, so the findings may not generalise to other health systems or cultural contexts. All study measures were self-report. Psychiatric diagnoses were reported by participants, but they were recorded by the psychiatrist who collected the data and could be checked against the clinical examination and available medical records; they were not, however, established by a structured diagnostic interview, and diagnostic composition was therefore not examined as a covariate. Trauma exposure and posttraumatic stress symptoms were not assessed, so the parallel drawn with cognitive models of acute and chronic trauma remains conceptual and should be tested directly. Self-injury status was based on a single forced-choice item whose “current” and “remitted” options overlap for individuals whose most recent episode occurred six to twelve months before assessment; because the date of the last episode was not recorded, some misclassification between these groups cannot be excluded. Assuming accurate self-report, participants who satisfied both definitions are most likely to have been classified into the current group, which may therefore include some individuals who had already stopped. The remitted group is less likely to be affected, although individuals with infrequent recent self-injury (on fewer than five days in the past year but within the past six months) fitted neither option exactly and could have selected either. This would attenuate differences between remitted participants and those who had self-injured in the past year. The lower suicidal ideation of remitted participants with a low-salience appraisal is therefore a conservative estimate, whereas the absence of a significant difference between remitted participants with a negative-dominant appraisal and participants who had self-injured in the past year should be interpreted with caution. Comparisons within the remitted group are less likely to be affected. The suppression effect observed for Shame is statistically robust in these data but conceptually unresolved and should not be acted on clinically until replicated. Finally, the outcome measure captures current suicidal ideation only; prospective data on attempts would be needed to establish predictive validity.

## 5. Conclusions

Cognitive appraisal of non-suicidal self-injury scars organises into three distinguishable and reasonably stable configurations with different clinical correlates: a low-salience profile, a negative-dominant profile associated with the greatest distress and suicidal ideation, and a strength-dominant profile associated with the strongest endorsement of the functions of self-injury. Appraisal is associated with suicidal ideation beyond current self-injury status and general distress, whereas the physical visibility of scars is not. Among patients whose self-injury has stopped, a persisting negative appraisal is accompanied by higher suicidal ideation than the other appraisal profiles, at a level that does not differ significantly from that of patients who have self-injured in the past year. Asking patients what they make of their scars may therefore be a useful part of routine assessment; whether appraisal profiles can guide the choice of intervention or the timing of discharge requires longitudinal and interventional evidence.

## Figures and Tables

**Table 1:** Sociodemographic and clinical characteristics of the sample, overall and by profile of scar appraisal (*N* = 234).

Characteristic	Total (*N* = 234)	Profile 1 (*n* = 129)	Profile 2 (*n* = 66)	Profile 3 (*n* = 39)	*p*
Age, years, M (SD)	24.34 (6.92)	25.59 (7.93)	22.76 (5.43)	22.90 (4.42)	0.005
Gender, *n* (%)					0.091
Female	174 (74.4)	88 (68.2)	52 (78.8)	34 (87.2)	
Male	49 (20.9)	34 (26.4)	10 (15.2)	5 (12.8)	
Not reported	11 (4.7)	7 (5.4)	4 (6.1)	0 (0.0)	
Education, *n* (%)					0.001
Primary	9 (3.8)	3 (2.3)	3 (4.5)	3 (7.7)	
Secondary	42 (17.9)	29 (22.5)	11 (16.7)	2 (5.1)	
Vocational secondary	67 (28.6)	38 (29.5)	20 (30.3)	9 (23.1)	
Higher	46 (19.7)	33 (25.6)	9 (13.6)	4 (10.3)	
Incomplete higher	70 (29.9)	26 (20.2)	23 (34.8)	21 (53.8)	
Occupation, *n* (%)					0.036
Employed only	80 (34.2)	50 (38.8)	19 (28.8)	11 (28.2)	
Studying only	60 (25.6)	22 (17.1)	23 (34.8)	15 (38.5)	
Employed and studying	39 (16.7)	22 (17.1)	9 (13.6)	8 (20.5)	
Neither	55 (23.5)	35 (27.1)	15 (22.7)	5 (12.8)	
Marital status, *n* (%)					0.964
Single	125 (53.4)	70 (54.3)	35 (53.0)	20 (51.3)	
Cohabiting	43 (18.4)	23 (17.8)	13 (19.7)	7 (17.9)	
In a relationship, not cohabiting	42 (17.9)	21 (16.3)	14 (21.2)	7 (17.9)	
Married	18 (7.7)	11 (8.5)	3 (4.5)	4 (10.3)	
Divorced	6 (2.6)	4 (3.1)	1 (1.5)	1 (2.6)	
Self-reported psychiatric diagnosis, *n* (%)					0.960
None reported	111 (47.4)	61 (47.3)	32 (48.5)	18 (46.2)	
Affective (depressive) syndrome	86 (36.8)	46 (35.7)	24 (36.4)	16 (41.0)	
Affective disorder with psychotic symptoms	21 (9.0)	14 (10.9)	5 (7.6)	2 (5.1)	
Personality-abnormal syndrome	12 (5.1)	6 (4.7)	4 (6.1)	2 (5.1)	
Self-injury status, *n* (%)					0.010
Current (past 12 months)	85 (36.3)	39 (30.2)	34 (51.5)	12 (30.8)	
Remitted (>6 months)	149 (63.7)	90 (69.8)	32 (48.5)	27 (69.2)	
Scar location, *n* (%)					0.048
Visible only	119 (50.9)	73 (56.6)	26 (39.4)	20 (51.3)	
Concealed only	71 (30.3)	40 (31.0)	21 (31.8)	10 (25.6)	
Both visible and concealed	44 (18.8)	16 (12.4)	19 (28.8)	9 (23.1)	

Note. Profile 1—low-salience appraisal; Profile 2—negative-dominant appraisal; Profile 3—strength-dominant appraisal. *p*-values: Kruskal–Wallis test for age, χ^2^ test for categorical variables. Psychiatric status reflects participants’ self-report of a previously communicated diagnosis and is not equivalent to a structured diagnostic assessment. “Affective disorder with psychotic symptoms” refers to psychotic features occurring within an affective episode; participants with schizophrenia spectrum disorders or a current psychotic episode were excluded.

**Table 2:** Scar appraisal, clinical measures and functions of self-injury by profile of scar appraisal (*N* = 234).

Measure	Profile 1 (*n* = 129) M (SD)	Profile 2 (*n* = 66) M (SD)	Profile 3 (*n* = 39) M (SD)	H	*p*	ε^2^
NSSI-SCS subscales						
Hopelessness	13.71 (3.81)	27.18 (7.71)	17.87 (6.28)	108.87	<0.001	0.463
Shame	9.22 (3.06)	19.53 (5.26)	10.62 (4.21)	110.01	<0.001	0.468
Inner Strength	7.75 (2.10)	8.44 (3.45)	17.85 (3.68)	95.37	<0.001	0.404
Clinical measures						
Suicidal ideation (BSSI)	14.91 (7.42)	19.58 (7.18)	15.72 (7.24)	15.52	<0.001	0.059
Social anxiety (LSAS)	107.10 (32.36)	135.15 (32.58)	113.95 (27.91)	26.91	<0.001	0.108
Body-image dysphoria (SIBID)	34.54 (21.01)	54.08 (18.01)	43.87 (18.73)	34.49	<0.001	0.141
Rumination (RRS)	43.84 (10.45)	54.73 (7.83)	48.49 (10.80)	44.34	<0.001	0.183
Functions of self-injury (ISAS)						
Intrapersonal domain	11.44 (5.94)	16.14 (4.02)	15.82 (4.87)	33.83	<0.001	0.138
Interpersonal domain	7.96 (6.26)	12.08 (7.65)	16.77 (10.28)	36.64	<0.001	0.150
Affect regulation	3.81 (1.98)	4.71 (1.30)	4.92 (1.33)	14.50	0.001	0.054
Self-punishment	3.12 (2.24)	4.80 (1.45)	3.87 (2.05)	26.02	<0.001	0.104
Anti-suicide	2.14 (1.98)	3.56 (1.87)	3.44 (2.46)	23.23	<0.001	0.092
Sensation seeking	0.70 (1.08)	0.88 (1.06)	2.18 (1.64)	33.92	<0.001	0.138
Toughness	1.15 (1.39)	1.68 (1.61)	2.54 (2.05)	16.62	<0.001	0.063
Autonomy	0.50 (1.05)	1.09 (1.74)	1.64 (2.27)	11.05	0.004	0.039
Marking distress	2.19 (1.83)	2.92 (1.95)	3.59 (1.86)	17.36	<0.001	0.066

Note. H—Kruskal–Wallis statistic (df = 2); ε^2^—epsilon-squared effect size. BSSI—Beck Scale for Suicide Ideation; LSAS—Liebowitz Social Anxiety Scale; SIBID—Situational Inventory of Body-Image Dysphoria; RRS—Ruminative Responses Scale; ISAS—Inventory of Statements about Self-Injury. NSSI-SCS—Non-Suicidal Self-Injury Scar Cognition Scale. Pairwise comparisons (Mann–Whitney U with Holm correction) are reported in the text.

**Table 3:** Proportion of participants endorsing key scar cognitions at least moderately, by profile (%).

NSSI-SCS Item	Total (*N* = 234)	Profile 1 (*n* = 129)	Profile 2 (*n* = 66)	Profile 3 (*n* = 39)
17. My scars make me feel like I want to kill myself	10.7	0.8	33.3	5.1
19. My scars make me feel like I could kill myself if I wanted to	35.9	17.1	69.7	41.0
14. My scars make me feel hopeless	19.2	3.1	57.6	7.7
22. My scars make me feel like I can’t change anything, like I’m stuck	27.4	9.3	66.7	20.5
6. My scars remind me that I am weak	38.0	26.4	72.7	17.9
8. My scars make me feel shame	34.6	20.9	69.7	20.5
10. My scars make me embarrassed in front of other people	39.3	20.2	83.3	28.2
23. I think that people judge me because of my scars	39.7	20.2	80.3	35.9

Note. Endorsement is defined as a rating of 3 (“moderately”), 4 (“strongly”) or 5 (“extremely”) on the 5-point NSSI-SCS response scale. Item numbers refer to the original 26-item NSSI-SCS. NSSI-SCS—Non-Suicidal Self-Injury Scar Cognition Scale.

**Table 4:** Hierarchical regression predicting suicidal ideation (Beck Scale for Suicide Ideation, BSSI) from scar appraisal (*n* = 230).

Predictor	B	SE (HC3)	β	*p*	Model R^2^	ΔR^2^
Step 1					0.133	—
Age	−0.234	0.094	−0.167	0.014		
Female gender	−0.357	1.146	−0.020	0.756		
Current self-injury	4.424	1.007	0.283	<0.001		
Visible scarring	1.334	1.029	0.081	0.196		
Step 2					0.262	0.129 ***
Rumination (RRS)	0.180	0.055	0.257	0.001		
Body-image dysphoria (SIBID)	0.003	0.032	0.007	0.935		
Social anxiety (LSAS)	0.043	0.018	0.189	0.016		
Step 3					0.341	0.079 ***
Hopelessness	0.353	0.070	0.374	<0.001		
Shame	−0.307	0.087	−0.244	<0.001		
Inner Strength	−0.128	0.086	−0.079	0.137		

Note. Coefficients shown for each variable are those of the model at the step at which the variable was entered. HC3—heteroscedasticity-consistent standard errors; RRS—Ruminative Responses Scale; SIBID—Situational Inventory of Body-Image Dysphoria; LSAS—Liebowitz Social Anxiety Scale; bootstrap 95% confidence intervals (5000 replications): Hopelessness 0.221 to 0.490, Shame −0.466 to −0.132, Inner Strength −0.295 to 0.040. *** *p* < 0.001. Final model: F(10, 219) = 11.35, *p* < 0.001; adjusted R^2^ = 0.311; all variance inflation factors ≤ 2.56.

## Data Availability

The anonymised dataset and the analysis scripts supporting the findings of this study are available from the corresponding author upon reasonable request; they will be deposited in a public repository upon acceptance. The data are not publicly available at present because they were collected from a clinical population under a consent procedure that did not include open publication of individual records.

## Supplementary Materials

The following supporting information can be downloaded at: https://www.mdpi.com/article/10.3390/ejihpe16100161/s1, Table S1: Stability of the three-profile solution (N = 234); Table S2: Covariate-adjusted comparisons of the appraisal profiles; Table S3: Diagnostics and sensitivity analyses of the hierarchical regression on suicidal ideation (n = 230).
