# Supplementary material for: Profiles of Cognitive Appraisal of Non-Suicidal Self-Injury Scars and Their Clinical Correlates in Adults with a History of Self-Injury: A Cross-Sectional Study
Source: Eur J Investig Health Psychol Educ. 2026 Oct 10;16(10):161. doi: 10.3390/ejihpe16100161 (PMC13703269; doi:10.3390/ejihpe16100161)
Supplement: Supplementary file 1 [file ejihpe-16-00161-s001.zip › ejihpe-4601040-supplementary.pdf]

Supplementary Materials

Profiles of Cognitive Appraisal of Non-Suicidal Self-Injury Scars and Their Clinical Correlates in Adults with a History of Self-Injury: A Cross-Sectional Study

Table S1. Stability of the three-profile solution (N = 234).

**Criterion**	**k = 2**	**k = 3**	**k = 4**	**k = 5**
Silhouette coefficient, k-means	0.403	0.416	0.348	0.349
BIC, Gaussian mixture (diagonal)	1859.1	1805.0	1800.5	—
Prediction strength (200 random splits)	0.87	0.71	0.50	0.40

**Cluster-wise bootstrap stability (1,000 resamples)**	**Mean Jaccard**	**5th percentile**
Profile 1 — low-salience (n = 129)	0.94	0.85
Profile 2 — negative-dominant (n = 66)	0.97	0.80
Profile 3 — strength-dominant (n = 39)	0.86	0.67

**Agreement**	**Adjusted Rand index**
Split-half: half-A centroids vs half-B own solution (500 splits)	median 0.82 (IQR 0.76–0.88)
Split-half: half-A centroids vs full-sample profiles	median 0.91 (IQR 0.85–0.95)
k-means vs Ward, k = 3	0.66
k-means vs Gaussian mixture, k = 3	0.47

**Three-profile solution × four-cluster k-means solution**	**4a low (n = 100)**	**4b severe negative (n = 32)**	**4c strength (n = 43)**	**4d intermediate negative (n = 59)**
Profile 1 (n = 129)	100	0	5	24
Profile 2 (n = 66)	0	31	0	35
Profile 3 (n = 39)	0	1	38	0
Cluster means: Hopelessness/ Shame/Inner Strength	12.9 / 8.2 / 7.6	32.7 / 22.1 / 10.1	16.9 / 10.1 / 17.1	20.3 / 15.8 / 7.4

Jaccard thresholds after Hennig (2007): ≥0.75 stable, ≥0.85 highly stable. Prediction strength after Tibshirani & Walther (2005). The four-cluster solution retains the strength-dominant profile and splits the negative-dominant profile by severity.

Table S2. Covariate-adjusted comparisons of the appraisal profiles.

Outcome	n	F (profile)	p	p (rank-based)	Partial R²	B P2 vs P1 (Holm p)	B P3 vs P1 (Holm p)	B P3 vs P2 (Holm p)
Suicidal ideation (BSSI)	230	5.51	0.005	0.008	0.049	3.69 (0.003)	1.01 (0.456)	-2.68 (0.124)
Social anxiety (LSAS)	234	14.75	<0.001	<0.001	0.121	25.96 (<0.001)	6.34 (0.310)	-19.61 (0.004)
Body-image dysphoria (SIBID)	234	19.54	<0.001	<0.001	0.146	17.93 (<0.001)	8.66 (0.041)	-9.26 (0.041)
Rumination (RRS)	234	25.36	<0.001	<0.001	0.156	9.47 (<0.001)	3.49 (0.100)	-5.98 (0.008)
ISAS intrapersonal	234	16.08	<0.001	<0.001	0.130	4.19 (<0.001)	3.85 (<0.001)	-0.34 (0.725)
ISAS interpersonal	234	14.23	<0.001	<0.001	0.144	4.22 (<0.001)	8.32 (<0.001)	4.10 (0.043)
Self-punishment	234	16.33	<0.001	<0.001	0.110	1.59 (<0.001)	0.75 (0.072)	-0.84 (0.053)
Sensation seeking	234	14.31	<0.001	<0.001	0.160	0.16 (0.355)	1.48 (<0.001)	1.32 (<0.001)
Toughness	234	7.06	0.001	0.002	0.078	0.53 (0.060)	1.31 (0.002)	0.78 (0.060)
Autonomy	234	7.44	<0.001	0.002	0.069	0.69 (0.010)	1.08 (0.016)	0.39 (0.394)
Marking distress	234	6.34	0.002	0.002	0.064	0.68 (0.055)	1.31 (0.002)	0.63 (0.128)

Linear models with HC3 standard errors adjusted for age, gender, education, occupational status, current self-injury status and scar visibility. F — Wald test of profile membership (2 df). B — adjusted difference in raw score units; Holm correction across the three contrasts within each outcome. Rank-based p from the same model fitted to rank-transformed scores. Remitted subgroup (n = 145), BSSI adjusted for the same covariates except self-injury status—profile F(2, 133) = 4.02, p = 0.020; P2 vs P1 B = 4.10, p = 0.012; P3 vs P2 B = −4.58, p = 0.015—versus currently self-injuring participants—remitted P2 B = −1.30, p = 0.392; remitted P1 B = −5.38, p <0.001.

Table S3. Diagnostics and sensitivity analyses of the hierarchical regression on suicidal ideation (n = 230).

Diagnostic	Result
Residual skewness/excess kurtosis	0.48 / 0.61
Shapiro–Wilk test of residuals	W = 0.984, p = 0.010
Breusch–Pagan test	LM = 22.31, p = 0.014 (addressed by HC3 standard errors)
RESET test (functional form)	F = 0.00, p = 0.944
Cook's distance, maximum; cases > 4/n	0.12; 12
Maximum |externally studentised residual|	4.32
Durbin–Watson	2.00
Variance inflation factors	all ≤2.56

Model	ΔR² Step 3	Hopelessness β (p)	Shame β (p)	Inner Strength β (p)

Original OLS (HC3)	0.079	0.374 (<0.001)	−0.244 (<0.001)	−0.079 (0.137)
Rank-transformed BSSI	0.085	0.386 (<0.001)	−0.253 (<0.001)	−0.087 (0.105)
Excluding 12 influential cases (Cook's D > 4/n)	0.134	0.485 (<0.001)	−0.307 (<0.001)	−0.060 (0.217)
Huber robust regression (B, unstandardised)	—	B = 0.393 (<0.001)	B = −0.352 (<0.001)	B = −0.108 (0.255)

Step 3 added the three NSSI-SCS subscales to age, gender, current self-injury status, scar visibility, rumination, body-image dysphoria and social anxiety.
